# Molecular detection of *Helicobacter pylori* in saliva of Sri Lankan adults with periodontitis, gastritis or both conditions

**DOI:** 10.1186/s12903-026-08421-4

**Published:** 2026-04-24

**Authors:** Maneesha Thathsarani Somarathna, Dhanushka Leuke Bandara, Sadaham Kaushika Viduthpriya Gunasekare, Lakshika Shamalie Nawarathna, Sulochana Wijetunge, Madhavi Priyanka Paranagama, Niluka Darshani Gunawardhana

**Affiliations:** 1 Department of Basic Sciences, Faculty of Dental Sciences, University of Peradeniya, Peradeniya, Sri Lanka; 2 Department of Oral Medicine and Periodontology, Faculty of Dental Sciences, University of Peradeniya, Peradeniya, Sri Lanka; 3 Department of Surgery, Faculty of Medicine, University of Peradeniya, Peradeniya, Sri Lanka; 4 Department of Statistics and Computer Science, Faculty of Science, University of Peradeniya, Peradeniya, Sri Lanka; 5 Department of Pathology, Faculty of Medicine, University of Peradeniya, Peradeniya, Sri Lanka

**Keywords:** *H. pylori*, Periodontitis, Gastritis, Saliva, Oral cavity, PCR, Sri Lanka

## Abstract

**Background:**

Periodontitis is an immuno-inflammatory disease affecting the tooth-supporting structures, primarily caused by dysbiosis of the oral microbiome. The involvement of one of the gastric pathogens, *Helicobacter pylori*, has been reported among individuals with periodontitis. However, the evidence regarding the association between oral *H. pylori*, periodontitis, and gastritis remains inconsistent and has not been investigated in Sri Lanka. Therefore, this study aimed to detect the oral *H. pylori* in a cohort of Sri Lankan adults and to evaluate its association with periodontitis and gastritis.

**Methods:**

This cross-sectional study recruited 214 adults from two tertiary care institutes in Sri Lanka. Participants were categorized into four groups: (A) periodontitis only (*n* = 60), (B) gastritis only (*n* = 51), (C) both periodontitis and gastritis (*n* = 48), and (D) healthy controls without periodontitis or gastritis (*n* = 55). Unstimulated saliva samples were collected, DNA was extracted, and *H. pylori* was detected using PCR targeting the *16S rRNA* gene. Positive samples were confirmed by *ureA* gene amplification. Detection rates were compared using Fisher’s exact test with Holm correction for multiple comparisons (*p* < 0.05).

**Results:**

*H. pylori* was detected in 44 of 214 participants (20.6%; 95% CI 15.4–26.5%): periodontitis only 20.0%, gastritis only 21.6%, both conditions 31.3%, and controls 10.9%. The highest detection rate was observed in individuals with both periodontitis and gastritis (31.3%; OR 3.71, 95% CI 1.31–10.55). Although this difference did not reach statistical significance after Holm correction (adjusted *p* = 0.084), it represents a biologically meaningful trend warranting further investigation with larger sample sizes. No significant associations were found with age or sex (*p* > 0.05).

**Conclusion:**

*H. pylori* DNA is detectable in the saliva of Sri Lankan adults, with the highest detection rate observed in individuals with both periodontitis and gastritis (31.3%). This clinically relevant trend suggests that the inflamed periodontium may provide a favorable niche for the pathogen, although larger studies are needed to confirm statistical significance. This study represents the first investigation in Sri Lanka to identify the oral cavity as a potential extragastric niche for *H. pylori* using salivary detection. However, PCR-based detection cannot distinguish viable colonization from transient contamination. Longitudinal studies with culture-based methods and viability testing are required to clarify whether the oral cavity serves as an active reservoir for *H. pylori* transmission and reinfection.

**Supplementary Information:**

The online version contains supplementary material available at 10.1186/s12903-026-08421-4.

## Introduction

Periodontitis is a multifactorial immuno-inflammatory disorder that develops through complex interactions between periodontal pathogens and the host’s immune responses [1]. It generally begins as gingivitis and may progress to irreversible destruction of the tooth-supporting structures, primarily characterized by gingival bleeding, periodontal pocket formation, and alveolar bone loss, leading to clinical attachment loss (CAL) [2]. If left untreated, periodontitis could result in tooth mobility and tooth loss, which impairs mastication and aesthetics, contributes to social disparities, and reduces quality of life [3].

According to the World Health Organization (2025), severe periodontal disease affects more than one billion people worldwide [4]. The primary factor contributing to this disease is dysbiosis of the oral microbiome. In addition to established periodontopathic bacteria such as *Porphyromonas gingivalis*, *Treponema denticola*, and *Tannerella forsythia*, increasing attention has been directed towards the possible presence of systemic pathogens in the oral cavity [5]. Among them, *Helicobacter pylori*, a major gastric pathogen, has drawn increasing attention in part because of its potential relevance to periodontal health [6].

*H. pylori* is a gram-negative, microaerophilic bacterium that specifically colonizes the pyloric region of the stomach in more than half of the world’s population [7]. It is known as the causative agent of a wide range of gastric pathologies, including chronic gastritis, peptic ulcer disease, and gastric malignancies. Due to its carcinogenic potential, the International Agency for Research on Cancer (IARC) has classified it as a Group 1 carcinogen [8–10].

Although the gastric mucosa is the primary niche for *H. pylori*, several studies have detected *H. pylori* DNA in saliva, dental plaque, and periodontal pockets [11–16]. This has led to competing hypotheses regarding the oral cavity’s role: it may serve as an extra-gastric reservoir harboring viable bacteria capable of causing gastric reinfection [11, 12], or oral detection may reflect transient contamination from gastroesophageal reflux, swallowed gastric contents, or passage through the oral cavity without true colonization. Distinguishing between these possibilities requires studies employing culture-based methods and longitudinal designs [14].

In these studies, the detection rates of *H. pylori* in oral cavities vary widely from 33% to 86%, largely due to differences in sample types, detection methods, and population characteristics [11–14]. The coexistence of *H. pylori* in both the oral cavity and the stomach suggests a possible association between oral and gastric diseases. This association is likely rooted in shared pathogenic mechanisms, such as bacterial persistence, immune modulation, and tissue destruction, which are characteristic of both *H. pylori*-induced gastritis and periodontitis [15]. Reflecting this possibility, numerous studies have reported higher oral *H. pylori* detection rates among individuals with periodontitis; however, the evidence remains inconsistent, and the nature of the association is still debated [13, 16, 17].

Despite compelling evidence from other regions, research on oral *H. pylori* in Sri Lanka is scarce. Existing Sri Lankan studies have primarily relied on gastric biopsies, serology, or dental plaque samples [18, 19]. Notably, in Sri Lanka, no published investigations have assessed *H. pylori* in saliva. While dental plaque is a recognized niche for the pathogen, saliva was selected as the primary diagnostic medium for this study because it provides a global representation of the entire oral cavity’s microbial environment rather than a site-specific one. Furthermore, salivary collection is easily obtainable, requires no specialized clinical skills for sampling, and offers a more practical, non-invasive model for large-scale epidemiological screening in resource-limited settings. Moreover, given the high burden of both periodontitis and gastritis in the country, together with the influence of population-specific factors such as traditional betel quid chewing, rice-based diet with spicy foods, and varying oral hygiene practices, socioeconomic conditions, and environmental exposures, locally generated evidence is essential for an accurate understanding of the oral-gastric *H. pylori* relationship. Traditional practices such as betel quid chewing, which is prevalent in Sri Lanka, may alter the oral microenvironment by inducing local tissue irritation, modifying salivary pH, and disrupting the oral microbiome composition, potentially creating a more favorable niche for *H. pylori* persistence. Additionally, dietary habits including the consumption of spicy foods may contribute to gastric irritation and increase the frequency of gastroesophageal reflux, potentially facilitating the transit of *H. pylori* from the stomach to the oral cavity. Therefore, this study was designed to fill this research gap by detecting *H. pylori* in saliva and evaluating its association with periodontitis and gastritis among a group of Sri Lankan adults.

## Materials and methods

### Study design and participants

This cross-sectional analytical study included 214 adults, recruited between February and August 2025, from patients attending the Periodontal Clinic of the Dental Teaching Hospital, Peradeniya, Sri Lanka and patients awaiting upper gastrointestinal endoscopy at the Teaching Hospital, Peradeniya, Sri Lanka.

Participants were categorized into four study groups based on a coordinated clinical evaluation of gastric and periodontal health.

Group A: Individuals with periodontitis and no gastritis.

Group B: Individuals with gastritis and no periodontitis.

Group C: Individuals with gastritis and periodontitis.

Group D: Individuals without gastritis and no periodontitis (Table 1).

Gastric status was evaluated by upper gastrointestinal endoscopy in symptomatic patients referred for clinical investigation of gastrointestinal complaints. Gastritis was confirmed by an experienced specialist based on endoscopic findings. Individuals who were asymptomatic and did not meet the clinical criteria for endoscopic referral were classified into the “no gastritis” group.

Periodontal status was assessed by a calibrated examiner using a standardized clinical protocol. Periodontal screening was carried out with basic periodontal examination, followed by a detailed periodontal examination in patients with code 4 in at least one of the sextants [20]. The 2017 World Workshop on the Classification of Periodontal and Peri-Implant Diseases and Conditions was used to diagnose periodontitis [21, 22]. Participants without clinical signs of periodontal disease on screening were classified as non-periodontitis.

Group D (healthy controls) consisted of individuals who were asymptomatic for gastric disease and showed no clinical evidence of periodontitis. To minimize selection bias, these participants were recruited from healthy volunteers and individuals accompanying patients to the clinics. Each control participant underwent the same rigorous screening process as the study groups to confirm their health status and ensure they were not visiting the hospital for systemic illnesses that could influence the oral microbiome. However, the absence of gastrointestinal symptoms does not exclude subclinical gastritis or asymptomatic *H. pylori* infection.

Of the 214 participants, 97 were males, and 117 were females, with a mean age of 47.5 ± 17.68 years, and none had received prior treatment for gastritis or periodontitis. All participants were recruited at the time of initial diagnosis to ensure they were treatment-naïve at the point of sample collection.

### Inclusion and exclusion criteria

Individuals aged 18 years or older with a minimum of 10 natural teeth (to allow adequate periodontal assessment) were eligible for inclusion. Participants were excluded if they were current smokers (tobacco use within the past 12 months), had diagnosed diabetes mellitus (Type 1 or Type 2, regardless of glycemic control) or other systemic conditions known to affect periodontal health (including autoimmune diseases, immunosuppressive therapy, or blood disorders) had used proton pump inhibitors within the preceding four weeks, had received antimicrobial therapy within the previous three months, were pregnant, or were receiving long-term non-steroidal anti-inflammatory drug therapy.

### Sample size calculation

The sample size was determined using a power analysis for the comparison of two independent proportions. Sample size was calculated based on previous studies [12, 23] reporting oral *H. pylori* detection rates ranging from 10% in healthy controls to 30–40% in individuals with periodontal disease, as no prior Sri Lankan salivary *H. pylori* data were available. To detect a 20% difference in detection rates between groups with 80% power and α = 0.05 (two-tailed), a minimum of 47 participants per group was required using Fisher’s exact test. The final sample of 214 participants (48–60 per group) exceeded this minimum requirement.

### Ethical considerations

The Ethical clearance for the study was obtained from the Ethics Review Committees of the Faculty of Dental Sciences, University of Peradeniya, Sri Lanka (ERC/FDS/UOP/I/2024/85). Written informed consent was obtained from all participants prior to enrolment.

### Collection of unstimulated saliva samples

Approximately 2–3 mL of unstimulated saliva samples were collected from participants between 8:00 AM and 12:00 PM to minimize circadian variation in salivary composition. The collection occurred before endoscopic or periodontal assessments. Participants were instructed to refrain from brushing, eating, drinking, or mouth rinsing for at least one hour before saliva collection. Saliva was then directly expectorated into new sterile graduated containers, which were immediately sealed, kept on ice, and stored at -20 °C until DNA extraction.

### Isolation of bacterial DNA

Genomic DNA was extracted from 500 µL of saliva following a previously described method [19]. Briefly, the saliva sample was centrifuged at 300 × g for 10 min to collect the cells. Then the supernatant was eliminated, and the pellet was washed twice with 0.9% saline solution. The pellet was suspended in 100 µL of 50 mM NaOH solution and boiled for 10 min. After neutralization with 14 µL of 1 M Tris (pH 7.5), samples were centrifuged at 14,000 × g for 2 min, and the resulting supernatant was stored at -20 ℃ until polymerase chain reaction (PCR) analysis.

### PCR detection of *H. pylori*

*H. pylori* DNA was detected using the primers JW22/JW23 (5’- CGT TAG CTG CAT TAC TGG AGA − 3’and 5’ - GAG CGC GTA GGC GGG ATA GTC − 3’) that targeted the *H. pylori*’s * 16S rRNA* gene [6]. The PCR was performed in a 15 µL total reaction volume containing 7.5 µL of 2x Promega GoTaq^®^ Green Master Mix (USA), 1 µL of both forward and reverse primers, 4 µL of template DNA, and 1.5 µL nuclease-free water. Amplification was performed on a MiniAmp™ Thermal Cycler (Thermo Fisher Scientific, USA) under the following conditions. Initial denaturation at 95 °C for 5 min, 35 cycles of denaturation at 94 °C for 1 min, annealing at 60 °C for 1 min, and extension at 72 °C for 1 min, with a final extension of 72 °C for 10 min. A positive control (*H. pylori* ATCC 43629) and a negative control (nuclease-free water) were included in each run.

### Confirmation of the *H. pylori* positive samples

Samples positive for *16S rRNA* were confirmed by PCR targeting the *ureA* gene using primers HPU1 (5’ - GCC AAT GGT AAA TTA GTT − 3’) and HPU2 (5’ - CTC CTT AAT TGT TTT TAC − 3’) [19]. The PCR was performed in a 15 µL total reaction volume containing 7.5 µL of 2x Promega Go Taq^®^ Green Master Mix (USA), 1 µL of both forward and reverse primers, 4 µL of template DNA, and 1.5 µL nuclease-free water. Thermal cycling conditions included initial denaturation at 94 °C for 4 min, followed by 35 cycles of denaturation at 94 °C for 1 min, annealing at 45 °C for 1 min, and extension at 72 °C for 1 min, with a final extension of 72 °C for 10 min.

PCR products were electrophoresed on a 2% agarose gel in 1XTAE (Tris-HCl, 0.5% EDTA, and Glacial Acetic Acid) containing ethidium bromide. A 100 bp DNA ladder (Promega, USA) was used as a molecular size marker, and the gels were visualized with a Gel Documentation System (VILBER Bio Print TX4, France).

### Statistical analysis

Statistical analyses were performed in IBM SPSS Statistics v25. The primary outcome was salivary *H. pylori* detection (PCR positive/negative). Overall differences in detection across the four study groups were assessed using Fisher’s exact test, which was selected because expected cell counts were less than five in multiple cells (specifically, cells for Group D positive = 6, expected = 3.2 under the null hypothesis; Group C negative = 33, expected = 38.1). Expected cell counts were calculated as (row total × column total) / grand total. Six pairwise group comparisons were performed using Fisher’s exact test with Group D (Periodontitis - / Gastritis -) serving as the reference group for odds ratio (OR) calculations. *p*‑values were adjusted for multiple testing using the Holm method, and both unadjusted and Holm‑adjusted *p*‑values are reported. Associations between *H. pylori* detection and demographic variables (age category: 18–45 years, 46–75 years; and sex) were evaluated with Fisher’s exact test. Results are presented as counts and percentages, and effect sizes are expressed as odds ratios with 95% confidence intervals. All tests were two‑tailed, and statistical significance was set at *p*-value < 0.05. As the number of *H. pylori*-positive events within individual groups was limited, multivariable regression was not performed to avoid unstable estimates from an inadequate events‑per‑variable ratio.

## Results

### Detection of *H. pylori* in saliva samples of different study groups

This study involved a total of 214 participants: 60 in Group A, 51 in Group B, 48 in Group C, and 55 in Group D (Table 1). All participants were between 18 and 72 years old. The detection of *H. pylori* DNA in saliva was analysed as a binary outcome (positive/negative), with individuals in groups B and C confirmed to have gastritis through endoscopy.

**Table 1:** Characteristics of study participants and *H. pylori* detection rates by study group

Groups	Total (*N*)	Mean age (In years)	*H. pylori* positive participants (%)
Group A- Periodontitis (+), Gastritis (-)	60	52.58 ± 12.79	12 (20.0%)
Group B- Periodontitis (-), Gastritis (+)	51	43.04 ± 19.84	11 (21.6%)
Group C- Periodontitis (+), Gastritis (+)	48	52.42 ± 14.53	15 (31.3%)
Group D- Periodontitis (-), Gastritis (-)	55	41.75 ± 20.08	6 (10.9%)

**Fig. 1:**
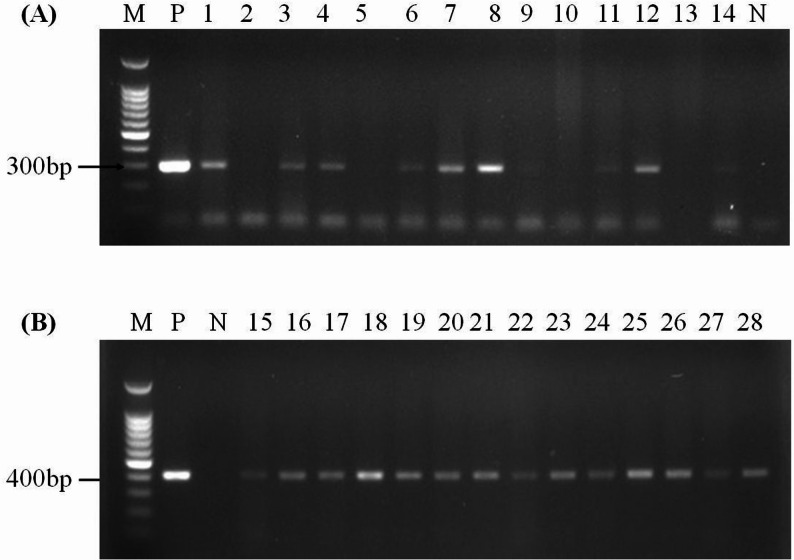
Agarose gel electrophoresis of PCR products from saliva samples. **A** For the JW22/JW23 primer pair (*16S rRNA* gene of *H. pylori*) showing an amplicon at 295 bp. **B** For HPU1/HPU2 primer pair (*ureA* gene of *H. pylori*) showing an amplicon at 411 bp. Lane M: 100 bp DNA Ladder; P: Positive control (*H. pylori* ATCC 43629); N: Negative control (Nuclease-free water); Lanes 1–28: representative saliva samples showing positive (clear band at expected size) and negative (no band) results

Overall, *H. pylori* DNA was detected in the saliva of 44 of 214 participants (20.6%, 95% CI 15.4–26.5%). Detection rates varied across groups, with the lowest rate in controls (Group D: 10.9%) and the highest in individuals with both periodontitis and gastritis (Group C: 31.3%). PCR analysis identified *H. pylori  16S rRNA* gene in 12 participants (20%) in Group A, 11 (21.6%) in Group B, 15 (31.3%) in Group C, and 6 (10.9%) in Group D with amplification bands observed at 295 bp (Fig. 1). A global comparison of detection frequencies across all four study groups using Fisher’s exact test yielded a *p*‑value of 0.053, approaching statistical significance and supporting the presence of an overall trend. Pairwise comparisons showed no statistically significant differences after Holm correction (*p* > 0.05), although the highest detection rate was observed in Group C (31.3%) (Table 2). Additionally, all positive samples for the *16S rRNA* gene were confirmed by the *ureA* gene of *H. pylori*, producing the expected 411 bp products.

**Table 2:** Pairwise comparisons of salivary *H. pylori* detection between study groups using Fisher’s exact test with Holm correction

Comparison	Odds Ratio	Lower 95% CI	Upper 95% CI	Holm adjusted *p*-value^†^
A vs. D	2.042	0.709	5.880	0.9310
B vs. D	2.246	0.764	6.606	0.9310
C vs. D	3.712	1.306	10.551	0.0843
A vs. B	0.909	0.362	2.280	1.0000
A vs. C	0.550	0.228	1.325	0.9310
B vs. C	0.605	0.245	1.495	0.9310

^†^Pairwise comparisons were performed using Fisher’s exact test.* p*-values were adjusted for multiple testing using the Holm method to control the family-wise error rate. Odds ratios (OR) with 95% confidence intervals (CI) are reported. All tests were two-tailed, and a *p*-value < 0.05 was considered statistically significant

Pairwise comparisons revealed that the experimental groups exhibited notably higher odds of salivary *H. pylori* detection compared to the control group (Group D). The most pronounced effect was observed in Group C (OR 3.71, 95% CI 1.31–10.55), representing participants with both periodontitis and gastritis. Although this comparison yielded a raw *p*-value of 0.014, the difference did not reach the threshold for statistical significance following the conservative Holm-Bonferroni correction (adjusted *p* = 0.084). We interpret this as a clinically relevant trend, not evidence of no association. A post-hoc power analysis based on the observed effect size (OR 3.71) showed that approximately 85 participants per group would be required to achieve 80% power. Our sample size of 48–60 per group was therefore underpowered, raising the possibility of Type II error (false-negative). This suggests that the absence of statistical significance may reflect limited sample size and conservative adjustment rather than a true lack of association. Larger, adequately powered studies are needed to confirm this clinically relevant trend.

Comparisons for Group A (OR 2.04) and Group B (OR 2.25) vs. Group D similarly showed increased odds but remained non-significant. The relatively wide confidence intervals observed across comparisons suggest that while a clear clinical trend exists, the observed effect sizes did not reach statistical significance following the stringent Holm-Bonferroni correction. Consequently, these results suggest a possible association that warrants further investigation in larger clinical cohorts.

### Association of *H. pylori* detection with the age and sex distribution

Of the 44 *H. pylori*-positive participants, 15 were male (15.46% of all males) and 29 were female (24.79% of all females). Detection was higher in participants aged 46–75 years (27/112; 24.1%) compared to those aged 18–45 years (17/102; 16.7%) (Table 3).

**Table 3:** *H. pylori* detection with age and sex

Study Group	Total (*N*)	Presence of *H. pylori* in saliva (%)	Age distribution of *H. pylori*-positive participants
A	60 Males-26 Females-34	12 (20%) Males-3 Females-9	18–45 yrs-3 46–75 yrs-9
B	51 Males-20 Females-31	11 (21.6%) Males-6 Females-5	18–45 yrs-5 46–75 yrs-6
C	48 Males-23 Females-25	15 (31.3%) Males-4 Females-11	18–45 yrs-6 46–75 yrs-9
D	55 Males-28 Females-27	06 (10.9%) Males-2 Females-4	18–45 yrs-2 46–75 yrs-4

Values are presented as a number (%). Associations between salivary *H. pylori* detection and demographic variables (sex and age category) were assessed using Fisher’s exact test. All tests were two-tailed, and a *p*-value < 0.05 was considered statistically significant

Associations between salivary *H. pylori* detection and demographic variables (sex and age category) were evaluated using Fisher’s exact test. No statistically significant associations were identified for either sex (sex vs. positivity: *p* = 0.114) or age category (age vs. positivity: *p* = 0.171).

## Discussion

### Detection of *H. pylori* in saliva

This study provides the first molecular evidence of *H. pylori* in the saliva of Sri Lankan adults, yielding an overall detection rate of 20.6% (95% CI 15.4–26.5%). Our findings are remarkably consistent with previous local data reported by Mallikaarachchi et al., who found a 19.7% detection rate among Sri Lankan university students [19]. This consistency across different age cohorts suggests that the oral cavity may be a stable extragastric reservoir for *H. pylori* within the Sri Lankan population. Internationally, our results fall within the established range of 21% to 38% observed in other regions [11, 22, 24]. Specifically, our detection rate is comparable to findings in Brazil (24%) [11] and the UK (21%) [23], while being higher than rates reported in Japan (6.4%) [25] and Iran (5.97%) [26]; yet significantly lower than the 65% reported in Saudi Arabia [27] or the 75% observed in China [28]. These geographic variations likely reflect differences in regional gastric prevalence, socioeconomic factors, and oral hygiene practices. Notably, the organism was identified in 10.9% of healthy controls, suggesting that oral carriage can occur independently of clinically evident periodontitis or gastric disease.

### Association with periodontitis and gastritis

A primary finding of this study is the strong numerical trend toward higher *H. pylori* detection in participants with concurrent oral and gastric inflammation. The highest detection rate was observed in individuals presenting with both periodontitis and gastritis (Group C, 31.3%). The odds of detection in this group were more than three times higher than in healthy controls (OR 3.71, 95% CI 1.31–10.55). Although statistical significance was not achieved after Holm-Bonferroni correction (adjusted *p* = 0.084), this likely reflects the conservative adjustment and limited sample size. We therefore emphasise this as a clinically relevant trend that warrants validation in larger, longitudinal studies. Participants with gastritis but without periodontitis (Group B) also demonstrated a two-fold higher detection rate (21.6%) than healthy controls. Similar findings have been reported in other populations [29], including a Korean study showing 28.6% positivity among patients undergoing endoscopy [30].

Furthermore, PCR-based studies from China have reported detection rates as high as 75% among individuals with biopsy-confirmed gastric infection [28]. These findings support the hypothesis that the inflammatory environment associated with both gastric and periodontal disease may provide a physiological niche that facilitates the persistence of *H. pylori* [12, 25]. The global Fisher’s exact test (*p* = 0.053) supports the presence of an overall trend across groups, and the lack of statistical significance in pairwise comparisons likely reflects the conservative nature of Holm correction and modest sample size rather than the absence of a biological connection.

### Methodological and demographic considerations

The use of PCR with validated JW22/JW23 primers ensured high analytical specificity, confirmed by 100% concordance with *ureA* gene detection [13, 31]. However, it must be acknowledged that PCR identifies bacterial DNA and cannot distinguish between viable, colonizing bacteria and transient DNA fragments. Oral detection could be influenced by gastroesophageal reflux or the swallowing of gastric contents, which may introduce bacterial DNA into the mouth without established colonization. Regarding demographics, detection was numerically higher in females (24.8%) and older participants (24.1%), though these associations were not statistically significant (*p* > 0.05). This suggests that local oral health and gastric status are likely more influential drivers of carriage than age or sex alone [32, 33].

### Clinical and epidemiological implications

The detection of *H. pylori* DNA across all groups, including healthy controls (10.9%), suggests that the oral cavity serves as an important extragastric reservoir in the Sri Lankan population. Oral persistence could potentially contribute to oral–oral transmission or gastric reinfection following eradication therapy, particularly in regions with moderate-to-high *H. pylori* prevalence. Our findings, alongside the 19.7% detection rate previously reported among Sri Lankan students [19], underscore the epidemiological relevance of the oral niche. While these strong clinical trends suggest that the oral cavity plays a role in the *H. pylori* infection cycle, further longitudinal evidence is required before salivary detection can be established as a standard clinical screening tool.

### Strengths and limitations

The major strength of this study is that it contributes to the limited evidence on oral *H. pylori* in the Sri Lankan population. By utilizing a patient-friendly and cost-effective salivary sampling method, this study demonstrates a feasible model for large-scale epidemiological screening. Furthermore, the use of dual-target PCR (*16S rRNA* and *ureA*) with 100% concordance provides high confidence in the specificity of our molecular detection.

However, certain limitations must be noted. The cross-sectional design prevents the determination of a causal or temporal relationship between oral carriage and gastric disease. Additionally, PCR detection of DNA does not confirm bacterial viability, meaning the findings could reflect transient DNA from gastroesophageal reflux rather than active colonization. Studies show that dental plaque often has higher *H. pylori* detection rates (up to 86%) than saliva (21–38%) [11, 12, 14], suggesting that biofilm may be a more stable niche. This difference likely reflects the structured polymicrobial environment of subgingival plaque, which may provide physical protection and microaerophilic micro-niches favorable for *H. pylori* persistence, compared to saliva, which is more susceptible to transient contamination from gastroesophageal reflux or passage of swallowed gastric contents. Whether our detection in saliva represents true oral colonization or transient contamination remains unknown. Moreover, the hospital-based recruitment and sample size may limit the generalizability of these findings to the broader community. Furthermore, because control participants were selected based on the absence of gastric symptoms rather than on confirmatory *H. pylori* testing (e.g., urea breath test or stool antigen test), some individuals in this group may have been asymptomatic *H. pylori* carriers. This could have reduced the observed differences between groups. Additionally, gastric *H. pylori* status was not assessed in participants; therefore, we cannot confirm whether oral detection reflects true colonization or correlates with gastric infection status.

### Future research directions

The findings of this study highlight several critical avenues for future investigation into the role of the oral cavity in the *H. pylori* infection cycle. A primary requirement is the implementation of viability assessments, utilizing culture-based methods or RNA-based detection, to distinguish active oral colonization from the presence of transient DNA fragments. To definitively test the extragastric reservoir hypothesis, future research should prioritize paired gastric and oral sampling combined with high-resolution strain genotyping. Such an approach is necessary to confirm whether oral and gastric *H. pylori* represent identical strains within the same individual, thereby establishing a clear pathway for transmission or reinfection. Additionally, future studies should incorporate paired sampling from both saliva and subgingival plaque within the same individuals. Since subgingival plaque represents a more structurally stable biofilm environment than saliva, comparative analysis of these two sample types would help clarify whether oral *H. pylori* detection in saliva reflects true colonization of the periodontium or transient contamination.

Beyond microbiological characterization, longitudinal cohort studies are warranted to track colonization stability over time and determine whether the persistence of oral *H. pylori* serves as a reliable predictor of gastric reinfection following successful eradication therapy. These efforts should be complemented by larger, age-matched investigations to enhance the precision of these molecular findings and establish more definitive associations in the broader Sri Lankan population. Based on the effect size identified in our post-hoc power analysis, future studies would require approximately 85 participants per group to definitively establish the complex associations between *H. pylori* carriage and the dual burden of periodontitis and gastritis. If the oral cavity is confirmed as a reservoir for *H. pylori*, periodontal therapy (scaling and root planing) combined with improved oral hygiene may help reduce oral bacterial load and potentially improve gastric eradication outcomes. Future studies should test whether adjunctive periodontal treatment reduces reinfection rates.

## Conclusion

This study establishes the first molecular baseline for *H. pylori* in the saliva of Sri Lankan adults, utilizing a rigorous dual-target PCR approach. This bacterium was detected in saliva at varying proportions across the cohort, including healthy individuals and those with periodontitis, gastritis, or both. Although the pairwise differences in detection rates did not reach statistical significance after conservative adjustments, the clinically relevant trend and effect size observed in patients with concurrent periodontitis and gastritis (OR 3.71) suggest that the inflamed periodontium may provide a favorable niche for the pathogen’s persistence. The persistence of *H. pylori* DNA in saliva supports the concept that the oral cavity may serve as a potential reservoir, contributing to transmission or gastric reinfection. Further longitudinal studies with culture-based viability testing are needed to elucidate the clinical relevance of oral *H. pylori* and its definitive role in transmission and reinfection.

## Supplementary Information

Supplementary Material 1.

## Data Availability

The datasets used and analyzed during the current study are available from the corresponding author on reasonable request. Full-length, unprocessed images of the original agarose gels have been provided as supplementary information to ensure data transparency.

## Abbreviations

DNA: Deoxyribonucleic Acid
rRNA: Ribosomal Ribonucleic Acid
PCR: Polymerase Chain Reaction
CAL: Clinical Attachment Level
PPD: Probing Pocket Depth
NaOH: Sodium Hydroxide
ATCC: American Type Culture Collection
TAE: Tris Acetate Ethylenediaminetetraacetic Acid
EDTA: Ethylenediaminetetraacetic Acid
SPSS: Statistical Package for Social Sciences
CI: Confidence Interval
OR: Odds Ratio
