# Supplementary material for: Molecular detection of Helicobacter pylori in saliva of Sri Lankan adults with periodontitis, gastritis or both conditions
Source: BMC Oral Health. 2026 Apr 24;26:1076. doi: 10.1186/s12903-026-08421-4 (PMC13281471; doi:10.1186/s12903-026-08421-4)
Supplement: Supplementary file 1 — Supplementary Material 1. [file 12903_2026_8421_MOESM1_ESM.pdf]

Supplementary File 1: Original Unprocessed Gel Images

Supplementary Figure 1: Original Unprocessed Gel Image for Figure 1 (For *H. pylori 16S rRNA* gene detection)

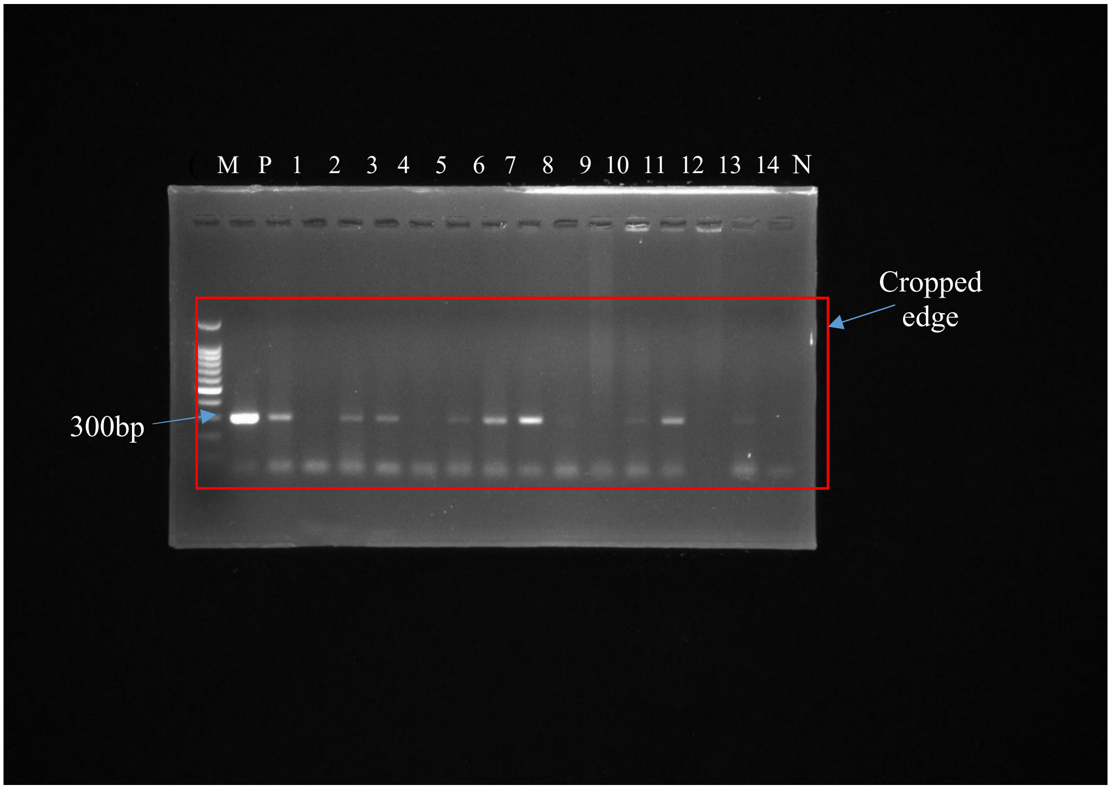

Supplementary Figure 1: Full-length, unprocessed agarose gel electrophoresis image used to generate Figure 1 in the manuscript. The image shows the PCR products of the *H. pylori 16S rRNA* gene with an expected size of 295 bp. The red box indicates the specific region cropped for the main manuscript. Lane M: 100 bp DNA Ladder; Lane P: Positive control (*H. pylori* ATCC 43629); Lane N: Negative control (Nuclease-free water); Lanes 1–28: Representative saliva samples.

Supplementary Figure 2: Original Unprocessed Gel Image for Figure 2 (for *H. pylori ureA* gene detection)

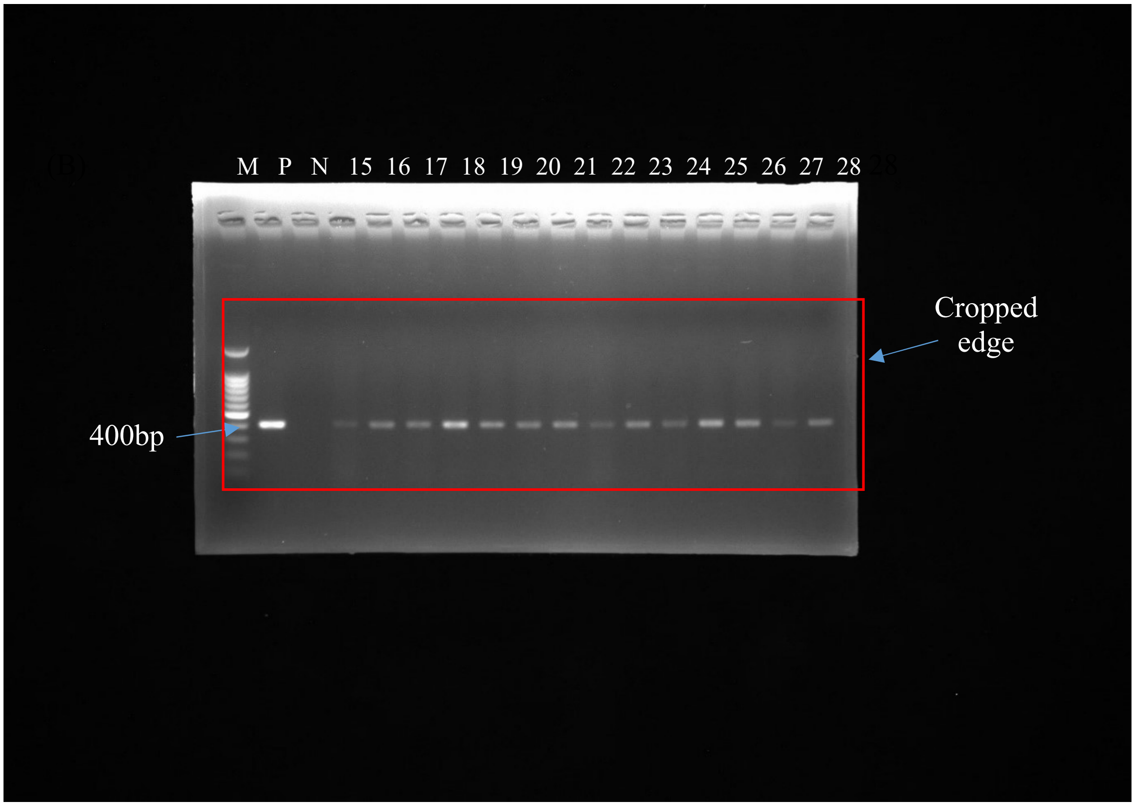

Supplementary Figure 2: Full-length, unprocessed agarose gel electrophoresis image used to generate Figure 2 in the manuscript. The image shows the PCR products of the *H. pylori* ureA gene with an expected size of 411 bp. The red box indicates the specific region cropped for the main manuscript. Lane M: 100 bp DNA Ladder; Lane P: Positive control (*H. pylori* ATCC 43629); Lane N: Negative control (Nuclease-free water); Lanes 1–28: Representative saliva samples.
